# Eye Behaviour in a Targeting Task in Children with ADHD: Linkage to a Level of Attention

**DOI:** 10.3390/bioengineering12111149

**Published:** 2025-10-23

**Authors:** Ondrej Jesina, Rudolf Psotta, Daniel Dostál, Ludvík Valtr

**Affiliations:** 1 Department of Adapted Physical Activities, Faculty of Physical Culture, Palacký University Olomouc, 771 11 Olomouc, Czech Republic; 2 Department of Wellness and Nutrition, College of Physical Education and Sport PALESTRA, 197 00 Prague, Czech Republic; psotta@palestra.cz; 3 Department of Psychology, Faculty of Arts, Palacký University Olomouc, 779 00 Olomouc, Czech Republic; daniel.dostal@upol.cz; 4 Department of Natural Sciences in Kinanthropology, Faculty of Physical Culture, Palacký University Olomouc, 771 11 Olomouc, Czech Republic; ludvik.valtr@upol.cz

**Keywords:** attention, ADHD, eye movements, visuomotor tasks, inhibition, fixation

## Abstract

Children with attention deficit hyperactivity disorder (ADHD) often exhibit different oculomotor behavior compared to their typically developing peers. Research shows that eye movement patterns can provide important information about attention mechanisms. While eye movements have been examined in various cognitive contexts, this study investigated their role in a task designed to assess their potential as indicators of attention functioning in children with ADHD. Specifically, we assessed tonic attention, attentional focus, and selective attention. Seventy participants aged 9–12 years with DSM-5 ADHD-I and ADHD-C types participated in our research. We then included the results of 57 participants in our study. We used the d2-R attention test and the Reaction alertness test to determine the specifics we were looking for. We used Eye Tracking Glasses (ETG) 2w to capture eye movements. The results show that quiet eye (QE) duration does not reliably predict visuomotor performance in this population. Our findings further suggest that in children with ADHD, the QE phase is not the primary period for acquiring visual information important for movement planning; rather, relevant information is gathered earlier in the process. Conversely, prolonged onset and duration of QE were associated with poorer attentional efficiency, suggesting that in ADHD, longer QE may reflect slower or less efficient cognitive processing rather than increased control.

## 1. Introduction

Attention deficit hyperactivity disorder (ADHD) is characterized by core symptoms of inattention, hyperactivity, and impulsivity [1,2]. These symptoms are closely linked to impairments in executive functions (EF), a set of higher-order cognitive processes including attention, inhibitory control, and working memory that enable goal-directed behavior [3]. Deficits in EF have been consistently reported in children with ADHD [4], suggesting that alterations in these mechanisms play a central role in the etiology of the disorder. Attention and inhibition are fundamental for cognitive flexibility, response selection, and adaptive self-regulation, and disruptions in these domains have been identified as common markers across neurodevelopmental disorders such as ADHD, autism spectrum disorder, and dyslexia [5,6]. Grandjean et al. [7] linked deficits in interference control and inhibition to inappropriate social behavior, indicating that such difficulties may extend beyond physiological adaptability to broader aspects of social functioning.

These impairments contribute to reduced self-regulation, expressed both socially and cognitively, limiting behavioral control, adaptability, and the suppression of irrelevant stimuli [8]. While reaction time (RT) and error-based indices are widely used to assess cognitive control [9,10,11,12,13,14], they provide only a partial description of attention deficits. More recent perspectives emphasize the integration of motor and cognitive control, proposing that oculomotor and postural measures can serve as sensitive indicators of underlying cognitive mechanisms.

Oculomotor control, in particular, has been identified as an area of specific difficulty, and monitoring eye movements provides a sensitive, quantifiable indicator of brain function in children with neurodevelopmental disorders [15]. Children with ADHD often show reduced fixation stability and impaired inhibition of automatic saccades when orienting to visual targets [16]. Bucci et al. [17] demonstrated that children with ADHD, dyslexia, and autism allocate fewer attentional resources to demanding oculomotor tasks, leading to poorer performance and reduced postural stability. Feifel et al. [18] found that untreated children with ADHD were unable to suppress blinking and microsaccades while awaiting visual stimuli, whereas medication normalized these behaviors. Recent evidence by Forbes et al. [19] indicates that children and adolescents with ADHD, autism, or comorbid ADHD–autism conditions present distinct patterns of oculomotor dysfunction, further underscoring the sensitivity of eye-movement measures in neurodevelopmental populations. In contrast, Sanchez et al. [20] reported no association between antisaccade direction errors and ADHD traits, suggesting a genetically mediated relationship between voluntary eye movement control and inattention symptoms. Despite such variability, oculomotor suppression has been proposed as a useful measure of impulsivity and inhibitory control in ADHD, particularly when elicited by non-central stop signals [21,22,23]. Meta-analytic evidence supports the idea that oculomotor inhibition (e.g., antisaccade tasks) differs between ADHD and controls (e.g., [24]).

Moreover, broader reviews of oculomotor deficits in ADHD confirm that children with ADHD are generally less precise in making eye movements and require more time to complete oculomotor tasks than controls [25] (cf. “Oculomotor deficits in attention deficit hyperactivity disorder” review).

Recent research also highlights several approaches that may improve attention and inhibitory control in neurodevelopmental populations, including cognitive training, neurofeedback, mindfulness-based interventions, and motor-cognitive programs targeting coordination and eye movement control [26]. In particular, a recent systematic review suggests that conversational AI chatbots may support executive function reinforcement, including attentional and inhibitory processes, by providing interactive cognitive training in everyday settings [27]. Eye-tracking paradigms provide a unique window into these processes by allowing the direct observation of attentional allocation, visual scanning, and inhibitory mechanisms. Monitoring eye movements thus represents a promising tool for both understanding and enhancing cognitive control in children with ADHD.

**Figure 1:**
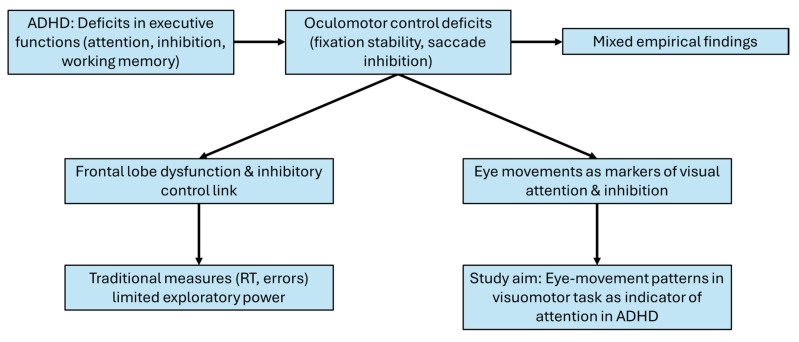
Conceptual Framework: Eye Movement and Attention in ADHD.

We consider oculomotor behavior to be a direct manifestation of visual attention—both covert and overt—and inhibitory control. Supporting this view, our previous findings [28,29] indicated that improved visuocognitive accuracy, particularly the selective suppression of distractors, contributed to enhanced concentration in a group trained with the Quiet Eye (QE) method. Building on these findings, a QE-based intervention emphasizing sustained fixation on a target during goal-directed hand–eye coordination tasks may strengthen the ability to allocate visual attention to relevant details within the visual field. Consistent with prior research, abnormalities in eye movement control among individuals with ADHD have been linked to dysfunctions in the frontal lobe [30]. More recent evidence highlights the interdependence of neural systems governing visual attention and oculomotor control, further supporting the notion that deficits in inhibitory control underlie attentional difficulties in ADHD [18]. Based on this framework, we hypothesize that oculomotor indices of inhibitory control can serve as reliable markers of attentional performance in children with ADHD. Specifically, patterns of eye behavior reflecting inhibitory control may provide measurable indicators from which attention deficits—and indirectly, ADHD symptomatology—can be inferred. A conceptual summary of the theoretical framework is presented in Figure 1. To test this hypothesis, we examined eye-movement variables during a goal-directed visuomotor task as potential predictors of attentional performance, which was simultaneously assessed using two standardized neuropsychological tests.

## 2. Materials and Methods

### 2.1. Participants

A total of 70 children aged 9–12 years who met the DSM-5 diagnostic criteria for either the inattentive subtype of ADHD (ADHD-I) or the combined subtype (ADHD-C) [31] were initially recruited for this study. However, only 57 participants (51 ADHD-I and 6 ADHD-C) were included in the final analysis. Thirteen children were excluded prior to data analysis: eight due to insufficient eye-tracking data quality (described below), two because of data loss caused by a camera battery failure during recording, and three who were unable to complete the full testing procedure and withdrew during the session. Participants were recruited from 12 public mainstream schools following the national Framework Educational Program for Elementary Schools. Children were excluded if they presented with severe anxiety, mood dysregulation, conduct disorder, intellectual disability, psychotic disorders, visual or hearing impairments, physical or neurological disabilities affecting motor function, uncorrected vision problems, or psychiatric medication use other than stimulant treatment (eligibility verified in collaboration with specialist physicians). Of the 57 participants, 11 were girls and 46 were boys; six were left-handed. Parents reported that 10 children were receiving stimulant medication (Ritalin or Concerta) to manage ADHD symptoms.

### 2.2. Ethical Approval and Informed Consent

All procedures were conducted in accordance with the Declaration of Helsinki (1975, and subsequent revisions). Written informed consent was obtained from participants’ legal guardians. Children were not informed of the specific aims of this study to avoid bias. The study protocol was approved by the Institutional Ethical Committee (approval code: 31/2018) and supported by the Science Foundation (project code: 19-18787S).

### 2.3. Study Design and Procedure

This study aimed to determine whether eye behavior during a target visuomotor task could serve as a valid indicator of attention levels in children with ADHD. Eye movement variables were analyzed as potential predictors of attention. Attention was assessed using two standardized neuropsychological tests. The attention tests (d2-R and Reaction Test of Alertness) were administered by two trained Ph.D. students with prior experience in standardized psychological testing. All assessments were conducted individually in a quiet classroom within the child’s regular school environment, free from distracting stimuli. Testing took place during morning hours (between 8:00 and 11:30 a.m.), aligning with the children’s usual school schedule to minimize variability in arousal levels that could affect reaction time performance.

### 2.4. d2-R Test of Attention

The d2-R Test of Attention is a paper-and-pencil measure of visual selective attention. Participants are presented with 14 rows, each containing 57 randomly mixed letters p and d, with one to four dashes above or below each letter. The target stimulus is the letter d with two dashes, while all other characters serve as distractors. Rows 2–13 include 308 targets and 376 distractors; performance in the first and last rows is excluded from scoring.

Participants are instructed to scan each row from left to right and mark the target characters as quickly and accurately as possible while ignoring distractors. Each row is timed for 20 s, resulting in a total test duration of 280 s.

Two variables were extracted for this study:

Concentration Performance (CP): a measure of focused attention, calculated as the number of processed targets minus commission errors (distractors marked as targets) presented as standardized scores (SS).

Percentage of Errors (%errors): a qualitative measure of accuracy, calculated as the total number of commission and omission errors expressed as a percentage of processed target characters presented as SS.

The d2-R Test was scored using age-adjusted normative data provided in the test software. The d2-R test demonstrates strong construct and convergent validity for assessing different aspects of focused attention (CP, %errors) [32]. Although our sample included children aged 9 to 12 years, reliability coefficients for the youngest subgroup (9–10 years) are not explicitly reported in the manual. Therefore, we refer to reliability values available for the closest age group. Test–retest reliability over one and ten days has been reported as very good to excellent for individuals aged 15–25 years (CP: 0.94 and 0.85; %errors: 0.84 and 0.47), and good to excellent for children aged 11–12 years in school settings [32].

### 2.5. Reaction Test of Alertness

The Reaction Test of Alertness (RTA), part of the Vienna Test System (VTS) (version 31; Schuhfried GmbH, Mödling, Austria), is a standardized computerized measure of attention. The test consists of two sets of 28 trials each. The first set presents a visual stimulus—a yellow circle (diameter: 3 cm)—without any preceding signal, while the second set involves an acoustic warning signal before the stimulus. For the purposes of this study, only the first set of non-signal trials was analyzed to assess tonic (intrinsic) attention.

In each trial, the stimulus appeared in the center of a black computer screen. Participants placed the index finger of their preferred hand on a home button (diameter: 2 cm) located 45 mm below a rectangular black response button (5.5 × 1.5 cm). Participants were instructed to press the response button as quickly as possible once the stimulus appeared, then return their finger to the home button. The stimulus remained on the screen until a response was recorded or a maximum of 1000 ms elapsed. Inter-stimulus intervals (ISI) varied between 2500 ms and 6500 ms.

The following variables were extracted:

Mean RT: indicator of tonic attention.

Intraindividual standard deviation of RTs (SDRT): measure of variability in attention.

Coefficient of variation of RTs (CVRT): measure of arousal regulation variability.

Number of correct responses (CorrResp).

The RTA demonstrates excellent reliability for RTs in non-signal trials (r = 0.965) [33].

### 2.6. Movement Task and Eye Behaviour Measurement

To assess eye behaviour during a motor task, participants performed the Catching with One Hand test from the Movement Assessment Battery for Children—Second Edition (MABC-2) [34]. Participants were instructed to throw a tennis ball against a wall and catch it with their preferred hand after the rebound, ensuring the ball did not fall to the ground. A line was placed 2 m from the wall, and participants were asked to stay behind it while throwing the ball, although they could cross the line when catching. Each participant completed five practice trials followed by 10 test trials according to the MABC-2 manual.

Eye behaviour was recorded using mobile Eye Tracking Glasses (ETG) 2w (SensoMotoric Instruments/SMI/, Teltow, Germany). The ETG was connected via cable to a mobile smart recorder (customized Samsung Galaxy S4) held by an instructor standing behind the participant. The ETG included a scene camera and a pair of infrared cameras. The scene camera recorded the participant’s forward view at 1280 × 960 pixels, 24 Hz, with a 46° vertical and 60° horizontal field of view. The infrared cameras detected pupil position and diameter at 60 Hz, with 0.5° accuracy for gaze position. The gaze tracking range was 60° vertically and 80° horizontally.

Before each recording, a three-point calibration was performed using three green squares stickers with a dimension of 1.5 × 1.5 cm positioned in a triangle shape 25 cm apart on a wall 2.5 m from the participant. Calibration accuracy was assessed by verifying that the gaze cursor overlapped with the area of each calibration point on the wall. Data collection did not begin until a successful calibration was achieved. Calibration failures were not recorded, as the experimenter repeated the calibration procedure until all three reference points were confirmed. Only trials with successful end-of-block calibration checks were saved and further processed. Consequently, all analyzed trials met the predefined calibration accuracy criterion.

In addition to ETG recording, arm movements were captured using an external camera (Panasonic HDC-TM900; 50 Hz, 14.2 MP), positioned 3.5 m lateral to the participant on the side of the throwing arm.

### 2.7. Eye Tracking Data Analysis

The SMI fixation detection algorithm implemented in BeGaze 3.7 software (SMI, Teltow, Germany) was applied to identify fixations. This proprietary algorithm determines fixations automatically based on velocity and dispersion thresholds. Details of a detection algorithm are not disclosed by SMI; however, the same software has been used for detecting eye events in previous studies [29,35,36,37]. Only fixation events were analyzed in the present study; blinks and saccades were excluded from all analyses. Gaze mapping to predefined areas of interest was not relevant in this case, as the task involved throwing toward a uniform white wall without marked target zones. Instead, fixations were quantified relative to the “virtual” target area defined by the location of the ball rebound. Trials with excessive data loss were excluded from analysis. Following recent recommendations, trials were considered unreliable if the tracking ratio was below 85% or the fixation ratio was below 60% [37]. Based on these criteria, data from 13 participants were excluded.

Prior to analysis, recordings from the ETG and external camera were time-synchronized. To synchronize the data of the eye behaviour record and the arm movement record, the time point was optically marked by a flash of light recorded simultaneously with the ETG scene camera and the external camera. Arm movements were analyzed using Dartfish 6.0 video analysis software (Dartfish, Fribourg, Switzerland),

Three critical moments of arm movement were identified in a frame-by-frame procedure:

Movement initiation. The moment of arm extension towards the target recognized as 1st frame when the elbow angle increased [38].

Ball release. 1st frame where dart was not in contact with the hand.

2000 ms prior to ball release. This moment was selected based on prior research indicating that this interval reliably captures the preparatory phase of goal-directed motor actions [39,40]. Specifically, studies have shown that movement initiation in throwing tasks typically occurs approximately 600 ms before release, meaning that the preparatory cognitive and perceptual processes begin around 1400 ms prior to release. By analyzing a 2000 ms interval, we ensured coverage of the entire preparatory phase, including early attentional engagement and motor planning. This approach is consistent with previous work using similar paradigms (e.g., Psotta et al., [29] and allows for a comprehensive assessment of gaze behavior leading up to movement execution.

Using the time-synchronized ETG and arm movement data, eye fixation data were extracted for each throwing trial using a custom algorithm implemented in C# (Microsoft. NET Framework 4.7, Microsoft Corp., Redmond, WA, USA). The following eye-tracking variables were analyzed:

Pre-throw QE duration: Defined as the last fixation within 1° of a “virtual” target on the wall before the initiation of arm movement. The operational definition QE used in this study was adopted based on established conventions in QE research (e.g., [41,42,43]). This threshold has been widely applied in studies investigating visuomotor control and attention, particularly in developmental and sport psychology contexts. The start of the final arm movement was operationalized as the frame in which elbow extension began from the initial 90° elbow angle [42,43].

QE onset: Calculated as the interval between the initiation of arm movement and the onset of the QE fixation.

Number of fixations: Total fixations within the 2000 ms pre-throw interval.

Total fixation duration (TT): Sum of durations of all fixations within the 2000 ms pre-throw interval (Figure 1).

This approach allowed for precise quantification of visual attention and gaze behaviour during critical phases of the throwing task.

### 2.8. Data Analysis

Data were analyzed using parametric statistical procedures. Normality of the variables was assessed with the Shapiro–Wilk test. Pearson’s product-moment correlation coefficient (r) and the coefficient of determination (R^2^) were used to examine the relationships between eye behaviour variables and attention test scores. Eye behaviour variables were considered potential predictors of attention performance. Due to strong intercorrelations among the eye behaviour variables, regression coefficients were not reported because they are highly unstable under these conditions.

To further explore the relationships between the set of attention variables and the set of eye behaviour variables, canonical correlation analysis (CCA) was performed. This method identifies pairs of correlated latent variables representing the two sets of observed variables. All analyses were conducted in R 4.3.0 using the CCA package [44] and the canonical correlation package (CCP) [45].

## 3. Results

The skewness of all variables examined ranged from −1 to 1, except for the variable CorrResp, which is significantly negatively skewed (−1.41). Although the Shapiro–Wilks test found a significant difference from a normal distribution in several variables (*p* < 0.05), given the sample size, this is not such a serious violation of the assumptions as to damage the reliability of the results, except for CorrResp. Descriptive statistics for each variable are summarized in Table 1.

Pearson correlation coefficients were calculated between each eye movement variable and the attention test scores. In addition, we calculated the percentage of shared variance between the eye movement variables and the corresponding attention test scale (i.e., the coefficient of determination). Significant difference of coefficients from zero was verified using the t or F statistic.

The used eye behavior variables can explain approximately 21% of the variance in the %errors variable (*p* = 0.016) and 16% of the variance in the CP variable of the d2-R Test of Attention (*p* = 0.055). For the VTS RTA test, only the CVRT variable showed a non-negligible association with the eye behavior variables, although this relationship is rather marginal (*p* = 0.082), in a similar manner to the CP. For a summary of the relationships between the variables, see Table 2.

**Figure 2:**
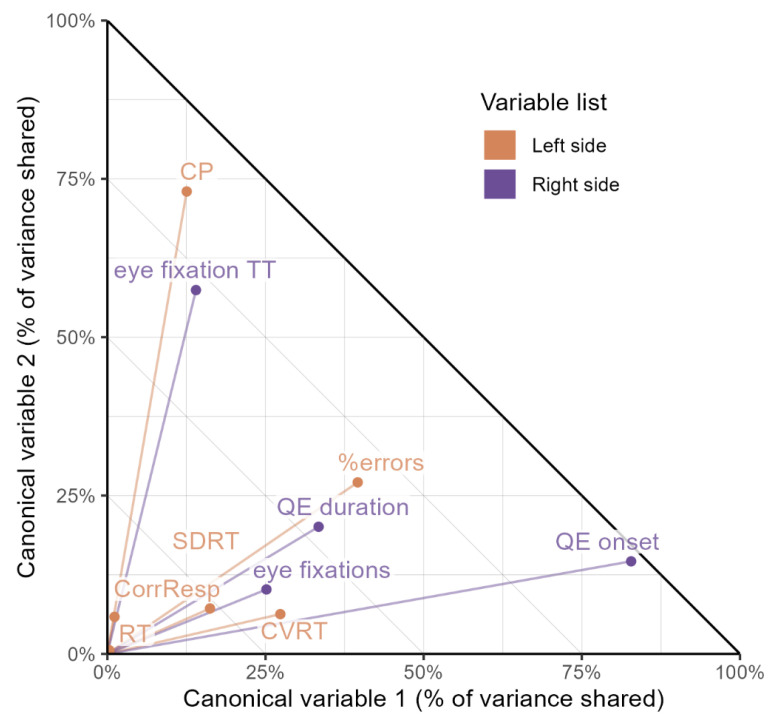
Graphical representation of canonical loadings. Note. The squared canonical loadings, i.e., the percentage of variance shared by the manifest variables and the corresponding canonical variables, are plotted on the axes of the graph. Since the canonical variables are orthogonal, the sum of squared correlation coefficients cannot exceed 100%, the plot therefore has the shape of a triangle.

The second way to explore the relationship between two groups of variables (the eye behavior variables—the variables of attention) is to analyze canonical correlations (see Figure 2). The first pair of canonical (latent) variables showed a correlation of 0.584. The permutation statistical test using Wilks lambda [45] found a *p*-value of 0.047, so we can conclude that strongly correlated latent variables are present in both groups of variables. After removing the first pair of canonical variables, the analysis found a second pair whose correlation is equal to 0.400. This coefficient no longer reaches statistical significance; given a *p*-value close to the required threshold (*p* = 0.081), let us examine it as well.

**Figure 3:**
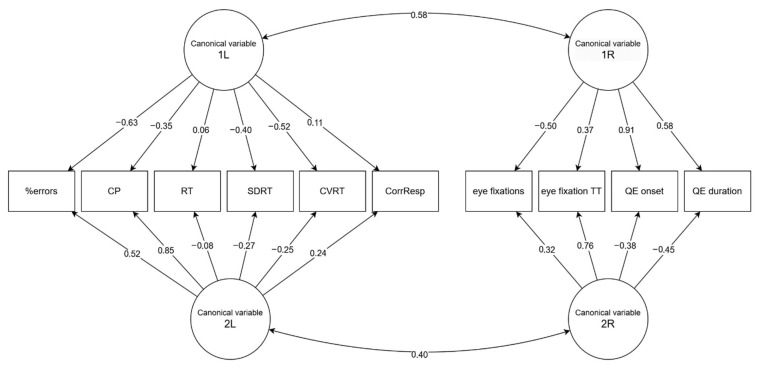
Canonical correlations—negative relations %errors a QE. CP—concentration performance. RT—reaction time. SDRT—standard deviation of RTs. CVRT—coefficient of variability for RTs. QE—quiet eye.

Regarding the first pair of canonical variables, we observed the strongest loadings in the group of predictors of the attention in QE onset (0.910) followed by QE duration (0.578) and eye fixations (−0.501). In the group of dependent attention scores, the variables %errors in the d2-R Test of Attention (−0.629) and CVRT in the VTS RTA (−0.523) dominated. This is consistent with our earlier observation—the strongest relationships were found between these variables. The second pair of canonical variables mainly concerns the eye fixations variable TT (0.758) on the right and CP (0.854) on the left side. A relationship independent on the first component should exist between these variables; however, let us add that, probably due to the limited strength of the second canonical correlation, this dependence is hardly noticeable in the matrix of Pearson correlation coefficients. See Figure 3 for a visual representation of canonical loading.

## 4. Discussion

The primary aim of this study was to examine whether eye-movement patterns can serve as reliable indicators of attentional functioning in children with ADHD. This rationale stems from the assumption that oculomotor behavior reflects underlying cognitive control processes, particularly inhibitory control, which is often impaired in ADHD. By analyzing eye movement variables during a goal-directed visuomotor task alongside standardized neuropsychological measures of attention, we sought to determine whether specific oculomotor parameters could predict attentional performance and, indirectly, ADHD-related symptomatology.

Previous research has consistently highlighted the role of oculomotor behavior as a window into attentional control processes. The quiet eye phenomenon, defined as the final fixation on a task-relevant location prior to movement initiation, has been widely associated with superior performance in visuomotor tasks, particularly in sports and high-pressure contexts [42]. Meta-analyses and systematic reviews confirm that longer QE durations typically distinguish experts from novices and correlate with successful performance outcomes [46]. However, findings in clinical populations, including children with ADHD, are less consistent. Some studies suggest that prolonged QE may reflect compensatory mechanisms or delayed processing rather than efficient attentional control [40]. Similarly, fixation patterns and gaze stability have been linked to inhibitory control, a core deficit in ADHD, as demonstrated by research using antisaccade paradigms [47]. Building on this theoretical framework, our study aimed to clarify whether these oculomotor markers can reliably predict standardized measures of attention in children with ADHD.

The canonical correlation analysis confirmed that oculomotor and attentional variables were systematically related. In the first canonical function, the strongest loadings were observed for QE onset (0.910), QE duration (0.578), and eye fixations (−0.501) on the predictor side, and for %errors in the d2-R Test (−0.629) and CVRT in the VTS–RTA test (−0.523) on the criterion side. The opposing signs of the loadings indicate that longer QE onset and QE duration were associated with lower standardized attention scores, reflecting poorer attentional efficiency but lower reaction-time variability, indicating a stabilizing effect on response consistency rather than a purely detrimental influence. Meanwhile, a higher number of fixations showed a weak positive association with attention scores, which may represent an alternative, compensatory visual strategy rather than efficient inhibitory control. The second canonical function, although statistically weaker, highlighted a distinct relationship between TT and CP. This suggests that fixation duration, independent of QE parameters, may serve as a more sensitive marker of attentional concentration. The fact that this relationship was not evident in simple Pearson correlations underscores the value of multivariate approaches like canonical correlation analysis in uncovering latent structures between behavioral domains.

The observed pattern suggests that prolonged QE onset and duration in children with ADHD may not reflect enhanced attentional control, as commonly assumed in neurotypical populations, but rather indicate slower or less efficient cognitive processing. Extended QE periods could represent an increased demand for visual information gathering and motor planning, possibly due to deficits in inhibitory control and attentional flexibility. This interpretation aligns with theoretical models of ADHD, which emphasize delayed or fragmented integration of sensory input into goal-directed actions. In contrast, the weak positive association of fixation frequency and total fixation duration with attentional measures may point to a compensatory strategy, where children attempt to stabilize performance by increasing visual sampling. Such a strategy, while adaptive, likely reflects less efficient visuomotor coordination compared to the streamlined processing observed in typically developing peers.

This study also suggests that the QE paradigm, although effective in enhancing visuomotor performance in athletes and the general population, may not be directly applicable to children with ADHD. In this population, earlier fixations may play a more critical role than the QE itself, possibly reflecting the need for a longer period to integrate visual information into motor planning. Evidence from our data supports this interpretation, as QE-related variables were negatively correlated with several behavioral indicators, %errors, CP, and the number of successful catches. These relationships indicate that longer QE durations QE onset were not associated with improved task performance, which stands in contrast to findings typically reported in neurotypical or athletic populations [48,49].

At present, there are no published studies confirming the validity or effectiveness of the QE concept in children with ADHD, suggesting that attentional and visuomotor control in this group may follow a different temporal pattern. This interpretation is consistent with our previous findings [29], where a training program based on the QE framework improved certain measures of attention and, in the ADHD group, enhanced throwing performance. However, no changes were observed in the core QE parameters (onset, offset, or duration), implying that these behavioral improvements likely occurred independently of QE-specific mechanisms.

In both the current study and the previous work by Psotta et al. [29], the total number of fixations within the 2000 ms pre-throw interval emerged as the most sensitive eye-behavior parameter. This convergence suggests that children with ADHD may rely on an earlier temporal window of visual processing to prepare motor actions. The positive correlation between the number of fixations and the number of successful catches found in the present study further reinforces this view, indicating that increased fixation frequency may reflect compensatory or less efficient visual sampling strategies.

Together, these findings point to the possibility that fixation dynamics—rather than the quiet eye itself—serve as more meaningful oculomotor markers of attentional functioning in ADHD. Understanding these earlier and more fragmented fixation patterns could provide valuable insights for designing targeted assessment tools and intervention strategies that better align with the unique visuomotor and attentional characteristics of children with ADHD. Nevertheless, some methodological limitations should be acknowledged when interpreting these results. A notable limitation of the present study concerns the sampling rate of the eye-tracking system. The ETG used in this study operated at a sampling frequency of 60 Hz, which restricts the temporal resolution of recorded eye movements. As a result, the precision of QE onset and duration measurements may be limited, particularly in fast visuomotor tasks. Moreover, this sampling rate precludes reliable detection and analysis of microsaccades. Future studies should consider using higher-frequency eye-tracking systems to enhance the temporal granularity of gaze behavior analysis and enable a more detailed investigation of fine-grained oculomotor events. Another potential limitation is the medication of some participants. However, for ethical reasons, medication was not discontinued and corresponds to reality. In the case of potential pedagogical work or diagnostic work in the system of school counseling facilities, medication will not be interrupted for the duration of the testing.

Building on the current findings, future research should aim to further clarify the temporal characteristics of gaze behavior in ADHD by combining high-resolution eye tracking with complementary neurophysiological measures. This multimodal approach could help identify the neural correlates of early fixation processing and determine whether these patterns reflect compensatory mechanisms or fundamental differences in visuomotor integration. Longitudinal and training studies would also be valuable to examine whether modifying fixation timing—rather than prolonging QE durations—can enhance motor performance and attentional control in children with ADHD. Such research could ultimately contribute to the development of evidence-based interventions tailored to the specific perceptual and cognitive needs of this population

## 5. Conclusions

The findings demonstrate that oculomotor variables, particularly fixation dynamics, are systematically related to standardized measures of attention. However, contrary to evidence from neurotypical and athletic populations, prolonged QE periods did not predict better performance. Instead, extended QE onset and duration were associated with poorer attentional efficiency, suggesting that in ADHD, longer QE may reflect slower or less efficient cognitive processing rather than enhanced control.

In contrast, fixation frequency and total fixation duration emerged as more sensitive markers of attentional functioning, likely representing compensatory strategies to stabilize performance. These results challenge the direct applicability of the QE paradigm to ADHD and highlight the need to consider alternative temporal patterns of visuomotor control in this population.

## Figures and Tables

**Table 1:** Descriptive statistics.

Scale	M (SD)	Five-Number Summary	Skewness	Shapiro–Wilk *p*-Value
***D2 Test of Attention***				
%errors (SS)	89.95 (11.03)	70–83–92–98–114	−0.18	0.147
CP (SS)	89.98 (9.64)	70–85–91–98–106	−0.42	0.036
***Reaction Test (VTS-RT)***				
RT (ms)	356.63 (54.44)	243–321–354–385–468	0.24	0.165
SDRT (ms)	55.86 (18.95)	20–41–52–68–102	0.54	0.048
CVRT (%)	15.47 (4.02)	8, 2–12, 7–15, 5–17, 8–25, 8	0.47	0.319
CorrResp (n)	26.58 (1.56)	21–26–27–28–28	−1.41	<0.001
***Eye movements***				
eye fixations (n)	3.95 (1.10)	1, 6–3–3, 9–4, 6–7, 9	0.67	0.085
TT (ms)	1474.82 (240.34)	908–1340–1448–1592–1995	0.09	0.848
QE onset (ms)	474.14 (226.73)	119–303–455–625–991	0.51	0.031
QE duration (ms)	754.49 (289.25)	167–561–792–891–1362	0.24	0.160

Note. Five number summary consists of minimum, Q1, median, Q3, and maximum. SD—standard deviation. SS—standard score. %errors—percentage of errors. CP—concentration performance. RT—reaction time. SDRT—standard deviation of RTs. CVRT—coefficient of variability for RTs. TT—Total fixation duration. QE—quiet eye.

**Table 2:** Pearson product moment correlation coefficients and coefficients of determination.

Scale	Eye Fixations (n)	Eye Fixations (Total Time)	QE Onset	QE Duration	Total Variance Shared (R^2^)
***D2 Test of Attention***					
%errors	0.383 **	0.082	−0.417 **	−0.332 *	0.206 *
CP	0.217	0.173	−0.323 *	−0.288 *	0.160
***Reaction Test (VTS-RT)***					
RT	−0.066	0.031	0.064	0.126	0.021
SDRT	−0.049	−0.215	−0.164	−0.039	0.094
CVRT	−0.030	−0.281 *	−0.242	−0.131	0.144
CorrResp	−0.056	0.077	0.024	0.013	0.018
***Catching performance***					
Catch (n)	0.197	0.222 *	−0.204	−0.320 *	0.184 *

Note. ** *p* < 0.01. * *p* < 0.05. The rightmost column shows the amount of unique variance of each scale which could be described using the QE and Eye fixation variables. Note. Statistical significance of Pearson correlations was double-checked using a nonparametric bootstrap. Bootstrap-based *p* values matched the conventional *t*-test results for all coefficients except the correlation between Catch (n) and Eye fixations (total time) variables, for which the robust *p* = 0.14. We therefore recommend interpreting this association with caution. CP—concentration performance. RT—reaction time. SDRT—standard deviation of RTs. CVRT—coefficient of variability for RTs. QE—quiet eye.

## Data Availability

Data are available on request due to privacy and ethical restrictions. The data presented in this study are available on request from the corresponding author.
